# Photoluminescence and Energy Transfer in Double- and Triple-Lanthanide-Doped YVO_4_ Nanoparticles

**DOI:** 10.3390/ma15072637

**Published:** 2022-04-03

**Authors:** Vassiliy A. Medvedev, Ilya E. Kolesnikov, Pavel K. Olshin, Mikhail D. Mikhailov, Alina A. Manshina, Daria V. Mamonova

**Affiliations:** 1 Institute of Chemistry, Saint Petersburg State University, Saint Petersburg 199034, Russia; st063851@student.spbu.ru; 2 Centre for Optical and Laser Materials Research, Research Park, Saint Petersburg State University, Saint Petersburg 198504, Russia; ilya-kolesnikov@mail.ru; 3 School of Natural science, Ulsan National Institute of Science and Technology, Ulsan 44919, Korea; pavel_olshin@bk.ru; 4 High School of Physics of Materials and Technologies, Peter the Great St. Petersburg Polytechnic University, Saint Petersburg 195251, Russia; mikhail.sudoma@yandex.ru

**Keywords:** oxide nanoparticles, luminescence kinetics, rare earth ions, codoped systems

## Abstract

Optical materials doped with several lanthanides are unique in their properties and are widely used in various fields of science and technology. The study of these systems provides solutions for noncontact thermometry, bioimaging, sensing technology, and others. In this paper, we report on the demonstration of YVO_4_ nanoparticles doped with one, two, and three different rare earth ions (Tm^3+^, Er^3+^, and Nd^3+^). We discuss the morphology, structural properties, and luminescence behavior of particles. Luminescence decay kinetics reveal the energy transfer efficiency (up to 78%) for different ions under the selective excitation of individual ions. Thus, we found that the energy transition from Tm^3+^ is more favorable than from Er^3+^ while we did not observe any significant energy rearrangement in the samples under the excitation of Nd^3+^. The observed strong variation of REI lifetimes makes the suggested nanoparticles promising for luminescent labeling, anticounterfeiting, development of data storage systems, etc.

## 1. Introduction

The combination of different types of rare earth ions (REI) in a single structure (single crystals, ceramics, glass ceramics, hybrid nanomaterials, etc.) represents a unique optical system, in which the luminescent properties can be tuned in a wide range by controlling the energy transfer between ions of different types through adjusting the composition and structure of the material. Such sophisticated optical systems can find applications in different fields, including ultrabroadband optical devices [1,2,3], microthermometry [4,5,6], bioimaging [7,8,9,10], and the production of light-emitting flexible materials [11,12,13].

To date, the optical properties of all rare earth ions are well studied, and luminescence properties of individual ions in different media are thoroughly described. However, the combination of several REI in one crystalline host can completely change the optical properties of the materials due to the energy transfer between the ions. Moreover, the luminescence behavior of these substances strongly depends on the types of REI, their concentrations, the type of the matrix, and the excitation mechanism. It is noteworthy that the effect of each of these parameters requires special consideration.

Metal oxides demonstrate several advantages for selection as the host for the REI doping. First, they are characterized by the stability of the physical and chemical behavior over a wide temperature range, biocompatibility, and mechanical hardness. The combination of these properties makes them promising candidates for a wide range of applications in mechanical engineering [14,15,16], medicine [17,18], sensing technology [19,20], anticounterfeiting [21,22], and others [23,24].

Many of these applications are based on the kinetic luminescence parameters that are unique for each specimen containing a different combination of ions. For example, luminescence coding indicators based on luminescence lifetimes, color spatial distribution, and intensity ratio between emission bands have been demonstrated to find multiple applications in different fields, such as bioimaging, deep tissue multiplexing labelling/detection, high-density data storage, and anticounterfeiting [25,26,27]. The temperature dependence of the kinetic parameters of oxide particles doped with REI can be used for luminescence nanothermometry [28,29].

Recently, we have reported on the synthesis and luminescence properties of YVO_4_:Tm^3+^, Er^3+^, and Nd^3+^ particles [30]. We selected these ions because of the absence of the overlapping luminescence bands, which makes it possible to study the direct energy transfer between the ions. In this paper, we demonstrate YVO_4_ nanoparticles doped not only with one or three but also with combinations of two different REI. Nanoparticles codoped with several ions were obtained thanks to adjusting the synthesis procedure—a modified Pechini method. The luminescence kinetics of the proposed optical system was studied in detail. Altogether, it provided a demonstration of the highly efficient energy transfer processes in codoped nanoparticles of YVO_4_ and a strong variation of REI lifetimes that is of great importance for luminescent labeling, anticounterfeiting, development of data storage systems, etc.

## 2. Materials and Methods

The particles were synthesized with the modified Pechini method that is described in detail in our previous paper [31]. Water solutions of yttrium and REI nitrates and vanadium oxide were used as initial reagents. Citric acid was chosen as the chelating agent to prepare a mixture of citrate complexes. Then, we added ethylene glycol to the solution to form a polymer gel with all metal ions within a cross-linked network. After this, we annealed the gel at 550 °C to decompose the complexes to the oxides (or complex oxides). It resulted in the formation of small particles that are characterized by a large number of defects. To solve this issue, we performed a high temperature (900 °C, 1 h) calcination of the mixture following the addition of KCl (the mass ratio 2:1 to the mass of the oxide particles) to the products of the previous stage of the reaction. This additional reaction step led to the formation of weakly agglomerated particles with a high degree of crystallinity. After the thermal treatment, we removed the potassium chloride with distilled water. 

Table 1 demonstrates the composition of the particles that we synthesized for further experiments. We prepared single-, double-, and triple-doped particles to thoroughly study the energy transfer between the ions and unravel its mechanism. The concentrations of each individual REI were selected separately and kept constant in all samples. It is known that increasing the REI concentration leads to a gradual increase of the luminescence intensity until concentration quenching starts, which results in a luminescence decrease at a higher concentration range of REI. This time, we increased the concentrations of the Tm^3+^ and Er^3+^ ions in the YVO_4_ samples to 1.0 and 3.0 at %, respectively, to enhance the luminescence intensity of these ions relative to the most intense emission band of the Nd^3+^ ions.

The structure of the samples was characterized with X-ray diffraction (XRD) using a Bruker “D8 DISCOVER” operating with a CuKα line (1.54056 Å). The crystal unit cell parameters and the coherent scattering region size were calculated using TOPAS software. The morphology of the powders was studied with scanning electron microscopy (SEM Zeiss Supra 40VP, Zeiss, Oberkochen, Germany); the size distribution of the particles in aqueous dispersions was analyzed with the static light scattering (SLS) method (Mastersizer 3000, Malvern Instruments Ltd, Malvern, Worcestershire, UK). The optical absorption spectra and luminescence properties of the samples were measured with a Lambda 1050 spectrophotometer and Horiba Jobin Yvon Fluorolog-3 spectrofluorimeter with a Xe-arc lamp (450 W power), respectively.

## 3. Results and Discussion

### 3.1. Structure and Morphology

**Figure 1:**
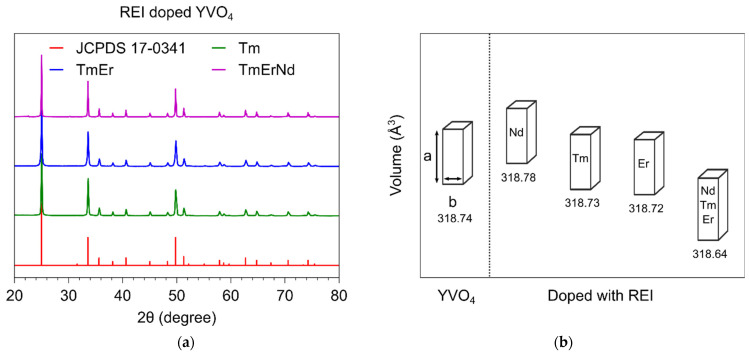
(**a**) XRD patterns and (**b**) unit cell volume of the YVO_4_ samples doped with different rare earth ions.

According to the X-ray diffraction analysis, all synthesized samples consist of an yttrium vanadate crystalline phase with a tetragonal structure (JCPDS 17-0341). Figure 1a shows several representative diffraction patterns of single-, double-, and triple-doped particles. The proper purification of the particles from potassium chloride after heat treatment in the salt melt is confirmed by the absence of KCl lines located at 28.3 and 40.5 degrees (JCPDS 04-0587). The coherent scattering region of all samples lies in a narrow range between 60–80 nm as the synthesis conditions were identical. However, the doping of the oxide crystalline host with the REI affects the unit cell parameters (Figure 1b) as the ionic radius of Y^3+^ (0.090 nm) is larger than that of erbium Er^3+^ (0.089 nm) and Tm^3+^ (0.088 nm) but smaller than the ionic radius of Nd^3+^ (0.098 nm). The smallest cell volume belongs to the triple-doped YVO_4_ as it contains an increased concentration of substitution ions with a smaller ionic radius (Er^3+^ and Tm^3+^).

**Figure 2:**
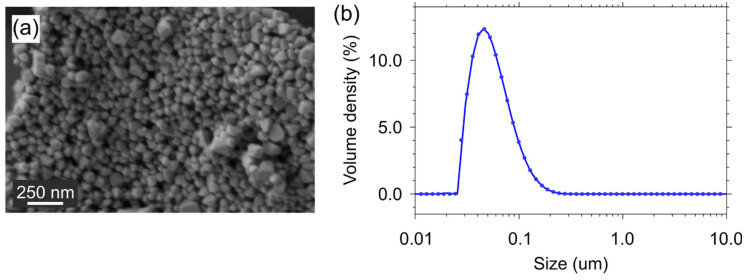
(**a**) SEM image and (**b**) size distribution of the triple-doped YVO_4_: Nd^3+^, Er^3+^, and Tm^3+^ particles.

Figure 2 demonstrates the morphology and the size distribution of the prepared particles. According to the SEM images (Figure 2a), the individual particles (with the size that rarely exceeds 100 nm) often possess an octahedral shape, which differs from that of the previously synthesized particles and is due to the higher volume of the salt melt (KCl) during the second stage of the heat treatment. This is also confirmed by the results of the SLS (Figure 2b) of the YVO_4_ powder in the aqueous solution. The experimental results propose a unimodal distribution of the particles with the average size of ~50 nm.

### 3.2. Luminescence Properties

**Figure 3:**
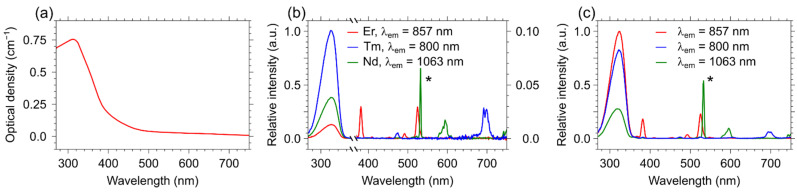
(**a**) Optical absorption spectra of pure YVO_4_ particles; (**b**) excitation spectra of the single-doped YVO_4_: Nd^3+^ (0.03 at.%), YVO_4_: Er^3+^ (3 at.%), and YVO_4_: Tm^3+^ (1 at.%) samples and (**c**) triple-doped YVO_4_: Nd^3+^ (0.03 at.%), Er^3+^ (3 at.%), and Tm^3+^ (1 at.%) samples monitored at different wavelengths λ_em_ = 1064, 857, and 800 nm corresponding to the luminescence emission bands of Nd, Er, and Tm, respectively. The asterisk (*) indicates the second order of diffraction.

The direct excitation of REI makes it possible to study the probability of energy transfer between different rare earth ions in the same host. For this purpose, the specific excitation wavelength for each REI has been chosen. Figure 3a presents an optical absorption spectrum of pure YVO_4_. The broad peak centered around 310 nm is attributed to the direct absorption of the YVO_4_ host, namely to the charge transfer from the oxygen ligands to the central vanadium atom inside the VO_4_^3−^ ion [32]. Excitation at this wavelength results in the simultaneous emission of all doping REI through the energy transfer from the crystalline host (Figure 3b,c) and significantly complicates the study of the energy transfer between ions. The excitation spectra were recorded at the most intense NIR emission lines of each dopant ion, 1064 nm, 857 nm, and 800 nm for Nd^3+^, Er^3+^, and Tm^3+^, respectively. From the obtained spectra, several lines for the selective excitation of ions of each type, ^4^I_9/2_ − ^4^G_5/2_ + ^4^G_7/2_ (595 nm) for Nd^3+^, ^4^I_15/2_ − ^2^H_11/2_ (526 nm) for Er^3+^, and ^3^H_4_ − ^3^F_2_ + ^3^F_3_ (692 nm) for Tm^3+^, were found.

**Figure 4:**
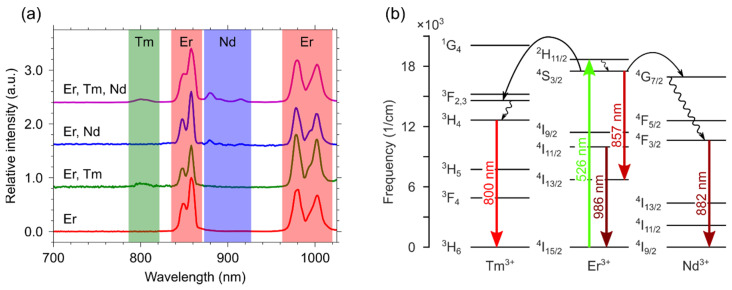
(**a**) Emission spectra and (**b**) scheme of energy migrations between ions for the single-, double-, and triple-doped YVO_4_: Nd^3+^ (0.03 at.%), Er^3+^ (3 at.%), and Tm^3+^ (1 at.%) samples under excitation with λ_ex_ = 526 nm.

Steady-state emission and luminescence kinetics are discussed individually for each ion that can act as a donor. Figure 4a shows the emission spectra of all samples containing erbium ions. The emission spectrum of the single-doped YVO_4_: Er^3+^(3 at.%) sample consists of ^4^S_3/2_ − ^4^I_13/2_ (857 nm) and ^4^I_11/2_ − ^4^I_15/2_ (986 nm) transitions. Codoping YVO_4_: Er^3+^ with another ion (Tm^3+^ or Nd^3+^) in the structure produces spectra with characteristic lines of both ions: ^3^H_4_ − ^3^H_6_ (800 nm) for Tm^3+^ and ^4^F_3/2_ − ^4^I_9/2_ (882 nm) for Nd^3+^. Since selective erbium excitation was used (λ_ex_ = 526 nm), this indicates an energy transfer between the active centres in the double-doped samples. The triple-doped sample demonstrates a simultaneous energy transfer from erbium to Nd^3+^ and Tm^3+^. From the analysis of the emission spectra for single-, double-, and triple-doped samples, we proposed an energy transfer scheme, which reflects the occurring processes upon the direct excitation of Er^3+^ ions (Figure 4b).

**Figure 5:**
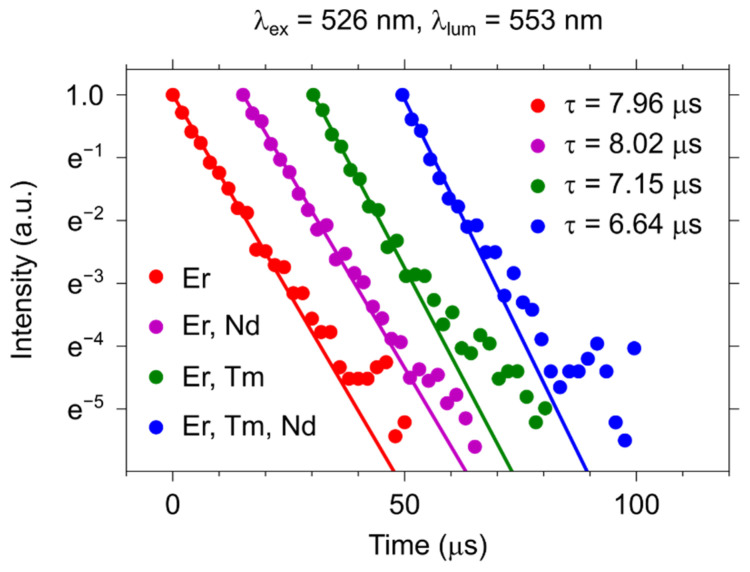
Luminescence decay curves of the one-, two-, and three-doped samples with the selective excitation of Er^3+^ ions.

Figure 5 shows the decay curves of erbium-containing samples monitored at the erbium ^4^S_3/2_ − ^4^I_15/2_ transition (λ_em_ = 553 nm, λ_ex_ = 526 nm). It is noteworthy that the luminescence decay curves started from 50 μs, but for the clear representation of the data, we shifted some datasets along the x-axis. The experimental data were fitted by a single exponential function: $I=I_{0}\cdot e^{-\frac{t}{\tau }\;}$, where τ is the observed lifetime of the ^4^S_3/2_ level. Due to the energy transfer in the double- and triple-doped samples, the luminescence lifetime decreases from 7.96 ± 0.13 µs (YVO_4_:Er^3+^) to 7.15 ± 0.15 µs (YVO_4_: Er^3+^, Tm^3+^) and 6.64 ± 0.20 µs (YVO_4_:Nd^3+^, Er^3+^, Tm^3+^). We do not observe a decrease in the lifetime for the YVO_4_: Er^3+^, Nd^3+^ sample due to the low concentration of Nd^3+^. 

The energy transfer efficiency (η) for the erbium-containing samples has been calculated using the following formula: $\eta =\left(1-\frac{\tau_{da}}{\tau_{d}}\right)\times 100\%$, where *τ_da_* is the donor lifetime in the presence of the acceptor and *τ_d_* is the unquenched donor lifetime (Table 2) [33,34]. In the double-doped Nd^3+^, Er^3+^ sample, the energy transfer efficiency is zero due to the low concentration of neodymium. However, this low concentration in the triple-doped sample gives a contribution to the energy transfer efficiency as this value in the double-doped Er^3+^, Tm^3+^ sample is lower (η = 10%) than in the triple-doped sample (η = 17%).

**Figure 6:**
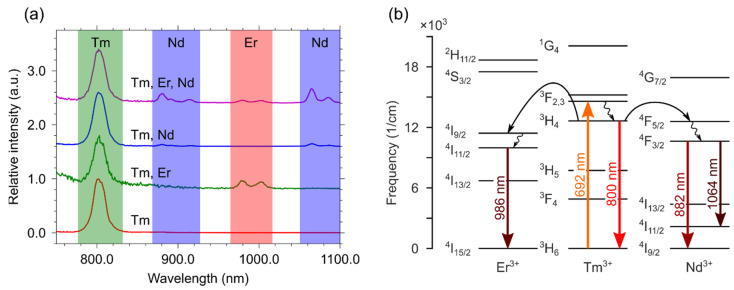
(**a**) Emission spectra and (**b**) scheme of energy migrations between ions for the single-, double-, and triple-doped YVO_4_: Nd^3+^ (0.03 at.%), Er^3+^ (3 at.%), and Tm^3+^ (1 at.%) samples under excitation with λ_ex_ = 692 nm.

Figure 6a displays the emission spectra of all samples containing thulium ions. The emission spectrum of the single-doped YVO_4_:Tm^3+^ (1 at.%) sample demonstrates a strong ^3^H_4_ − ^3^H_6_ (800 nm) transition. The introduction of the second ion (Er^3+^ or Nd^3+^) in the structure leads to the appearance of characteristic lines of the corresponding ion, namely ^4^S_3/2_ − ^4^I_13/2_ (857 nm) and ^4^I_11/2_ − ^4^I_15/2_ (986 nm) transitions for Er^3+^ and ^4^F_3/2_ − ^4^I_9/2_ (882 nm) and ^4^F_3/2_ − ^4^I_11/2_ (1064 nm) transitions for Nd^3+^. Since we selectively excite (λ_ex_ = 692 nm), this indicates a strong energy transfer between the active ions in the double-doped samples. Similarly, the triple-doped sample shows emission bands attributed to all three ions. Based on the analysis of the luminescence spectra for single-, double- and triple-doped samples, we suggest an energy transfer scheme following the direct excitation of Tm^3+^ (Figure 6b).

**Figure 7:**
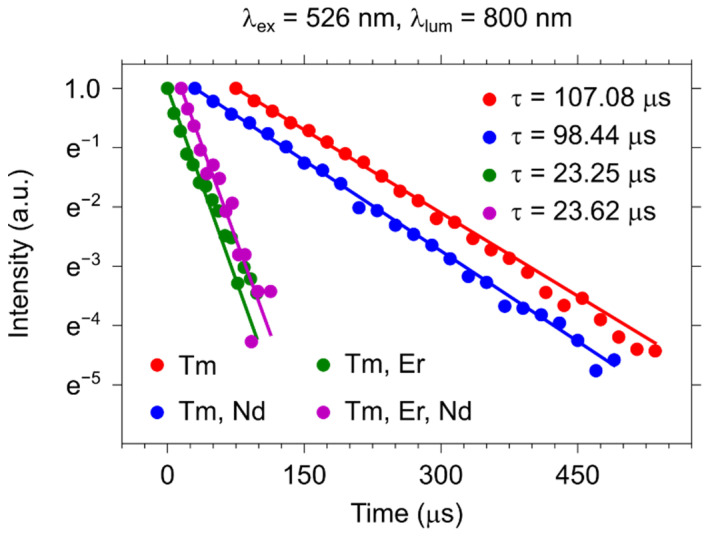
Luminescence decay curves of one-, two-, and triple-doped samples with the selective excitation of Tm^3+^ ions.

In Figure 7, we show the luminescence lifetimes of the YVO_4_ particles doped with Tm^3+^ and other REI monitored at the thulium ^3^H_4_ − ^3^H_6_ transition (λ_em_ = 800 nm) under direct excitation (λ_ex_ = 692 nm). Unlikely to the Er^3+^ lifetime, the addition of a small fraction of Nd^3+^ ions leads to a noticeable variation in the ^3^H_4_ lifetime (~9%). However, the presence of Er^3+^ ions in the double- and triple-doped samples drastically accelerates the quenching of the transition, and the lifetime reaches almost 23 µs for both cases. This fact is probably explained by a low energy mismatch of the Tm^3+^ and Er^3+^ excited levels, which results in an efficient Tm^3+^ − Er^3+^ energy transfer.

Table 3 has the values of energy transfer efficiency for the thulium-containing samples. In the double-doped Nd^3+^, Tm^3+^ nanoparticles, the efficiency reaches 8%. The sample containing Tm^3+^ and Er^3+^ ions showed the highest values of energy transfer efficiency (η = 78%) among the studied compositions.

**Figure 8:**
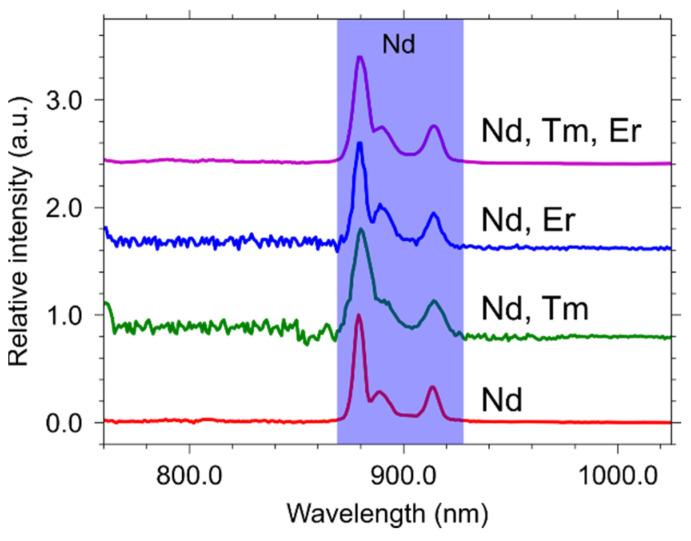
Emission spectra for the single-, double-, and triple-doped YVO_4_: Nd^3+^ (0.03 at.%), Er^3+^ (3 at.%), and Tm^3+^ (1 at.%) samples under excitation with λ_ex_ = 595 nm.

The emission spectrum of the single-doped YVO_4_: Nd^3+^(0.03 at.%) sample displays peaks assigned to the ^4^F_3/2_ − ^4^I_9/2_ transition (882 nm). The same emission lines are observed in the double- and triple-doped samples (Figure 8). The low concentration of neodymium in combination with the low energy transfer efficiency could explain the absence of erbium and thulium lines in the emission spectra when neodymium is directly excited.

## 4. Conclusions

In this work, we studied the energy transfer in nanocrystalline yttrium vanadate particles doped with different combinations of three ion types (Er^3+^, Tm^3+^, Nd^3+^). The nanoparticles were synthesized by the modified Pechini method. The powders are characterized by a high degree of crystallinity with an average size of the CSR in the range of 60–80 nm with the majority of the particles below 100 nm. The powders have weak agglomeration and can be transferred into a water solution. Luminescence spectra of all samples were studied upon the direct excitation of each ion (λ_ex_ = 526 nm, 692 nm, and 595 nm for Er^3+^, Tm^3+^, and Nd^3+^, respectively). From the analysis of characteristic emission lines, we determined the energy transfer between the different ion types in the double- and triple-doped samples. The luminescence kinetics are presented for the group of Er-containing samples and the group of Tm-containing samples under direct excitation into absorption bands of Er^3+^ and Tm^3+^, respectively. We found that the thulium ion displays a more efficient energy transfer than the erbium ion. The efficiency increases with the appearance of the third doping ion as an additional pathway for the donor’s energy transfer. The small energy transfer efficiency in binary systems with neodymium as the second ion are caused by the low concentration of this ion—a small number of ions causes the same low probability of energy transfer to them, and the high luminescence of neodymium ions ensures the presence of characteristic emission lines in the luminescence spectrum even at such low concentrations.

The demonstrated variability of the luminescence spectra and decay lifetimes at different excitation wavelengths for double- and triple- doped nanoparticles is promising for nanothermometry and systems of spectral encoding based on the ratio of different optical parameters. In such a way, nanoparticles of a simple structure, chemical composition, and morphology demonstrate the advantage in comparison with complicated core−shell multilayer structures.

## Figures and Tables

**Table 1:** Compositions of the crystalline particles.

№	Combinations of Rare Earth Ions	Concentration, at.%		
		Tm	Er	Nd
1	YVO_4_:Nd^3+^	-	-	0.03
2	YVO_4_:Er^3+^	-	3.0	-
3	YVO_4_:Tm^3+^	1.0	-	-
4	YVO_4_:Tm^3+^, Er^3+^	1.0	3.0	-
5	YVO_4_:Tm^3+^, Nd^3+^	1.0	-	0.03
6	YVO_4_:Er^3+^, Nd^3+^	-	3.0	0.03
7	YVO_4_:Tm^3+^, Er^3+^, Nd^3+^	1.0	3.0	0.03

**Table 2:** Values of energy transfer efficiency for one-, two-, and triple-doped systems with the selective excitation of Er^3+^ ions.

	Combination of Er^3+^ Ions		
	Er^3+^, Tm^3+^, Nd^3+^	Er^3+^, Tm^3+^	Er^3+^, Nd^3+^
η, %	17	10	0

**Table 3:** Values of energy transfer efficiency for the one-, two-, and triple-doped systems with the selective excitation of Tm^3+^ ions.

	Combination of Tm^3+^ Ions		
	Er^3+^, Tm^3+^, Nd^3+^	Er^3+^, Tm^3+^	Tm^3+^, Nd^3+^
η, %	78	78	8

## Data Availability

Not applicable.
